# College and Society Notes

**Published:** 1899-01

**Authors:** 


### COLLEGE AND SOCIETY NOTES.

### VETERINARY DEPARTMENT OF McGILL UNIVERSITY.

The alumni of the department will hold a reunion and dinner at the college hall, 6 Union Avenue, Montreal, on Saturday evening, February 4, 1899. Every graduate of the old school (Montreal Veterinary College, and now the Veterinary Department of McGill University) is most cordially invited to come and talk over old times, see old faces, renew your allegiance to your college parents, and give them by your presence renewed interest and courage in their work. The Secretary, Dr. John M. Parker (class of 1889) of Haverhill, Mass., is anxious to reach by circular letter every alumnus of the college, and solicits a communication from any who may not have been reached.

### ASSOCIATION MEETINGS IN JANUARY.

Illinois Veterinary Medical and Surgical Association, at the office of S. H. Swain, V.S., Decatur, Illinois, January 12th and 13th.

Iowa State Veterinary Medical Association at Des Moines, Iowa, January 10th and 11th.

### SOCIETY NOTES.

The Washington Veterinary Association is conducted in connection with the United States College of Veterinary Surgeons, and meets every second Wednesday.

Student societies are maintained at the Western Veterinary College and the Veterinary Department of the University of California. The latter department is contemplating an early occupancy of new buildings with greater facilities.

Harvard Veterinary Department Students' College Association meets weekly.

The Christmas examinations in connection with the Ontario Veterinary College were concluded on Friday, December 23d, when the following gentlemen passed a satisfactory examination and received diplomas: William A. Campbell, Niagara Falls,

South, Ont.; Arthur E. Chandler, Barberton, Ohio; Alexander J. R. Cromwell, Sawyerville, Quebec; William L. Crone, Waterford, Ontario; William Cunningham, Ashton, R. I.; John Hewines, Mountsberg, Ontario; Robert Lawson, Shoal Lake, Manitoba; Howard S. McFatridge, Halifax, N. S.; Donald H. McKay, Brandon, Manitoba; Cranston Owens, Utica, N. Y.; Philip C. Palmer, Bryn Mawr, Pa.; Harry P. Reed, Hemlock, N. Y.; Charles B. Shaw, Ashby, Mass.; and Winfield S. Wallace, Vermilion, N. Y. Primary examination: In anatomy, Elliott H. James.

The December meeting of the Keystone Veterinary Medical Association was one of the largest and most interesting ever held by the local society in Philadelphia.

The annual catalogue of the Louisiana State University and Agricultural and Mechanical College is one of the most artistic and complete to reach the editorial office this year. It indicates a progressive spirit in the educational field by the people of the "Pelican State" that is good to know. The work at the college, the experiment station, and farm indicates a live interest in the welfare of those in the State engaged in the various branches of agriculture and the livestock industry.

Dr. W. H. Dalrymple, instructor of veterinary science, gives much of his time to this work and unlimited attention to that of controlling and eliminating those contagious and infectious animal diseases peculiar to the climate and soil. Students taking the course in agriculture are given general instruction in those subjects relating to veterinary science, while those taking the sugar course, receive special instruction, covering the care, feeding, and management of farm animals and general hygiene. A commodious infirmary and pharmacy are among the facilities for instruction.

The approaching silver anniversary of the American Veterinary College in 1900 is meeting a hearty response among the alumni of that institution.

The proper feeding of well-bred range-colts will be tried expermentally this winter at the Iowa Agricultural College.

---

The smallest cows in the world are to be found in the Samoan Islands.

